# Predictors of increasing injury severity across suspected recurrent episodes of non-accidental trauma: a retrospective cohort study

**DOI:** 10.1186/s12887-016-0540-y

**Published:** 2016-01-16

**Authors:** Jonathan Thackeray, Peter C. Minneci, Jennifer N. Cooper, Jonathan I. Groner, Katherine J. Deans

**Affiliations:** Division of Child and Family Advocacy, Nationwide Children’s Hospital, Columbus, OH USA; Center for Surgical Outcomes Research and Center for Innovation in Pediatric Practice, The Research Institute at Nationwide Children’s Hospital, 700 Childrens Drive, JWest - 4th floor, Columbus, 43205 OH USA; Department of Surgery, Nationwide Children’s Hospital, Columbus, OH USA

**Keywords:** Non-accidental trauma, Child abuse, Injury, Recurrence

## Abstract

**Background:**

Little is known about how the severity of injury changes with recurrent events of suspected non-accidental trauma (NAT). Our objective was to determine risk factors for escalating severity of injury in children with multiple events of suspected NAT.

**Methods:**

This retrospective longitudinal cohort study included children from a pediatric Medicaid accountable care organization with ≥ 1 non-birth related episode containing an International Classification of Diseases, Ninth Revision, Clinical Modification or Current Procedural Terminology code for NAT or a skeletal survey between 2007 and 2011. Subsequent potential NAT events were defined as independent episodes with codes for either NAT, a skeletal survey, or injuries suspicious for abuse. Severity of injury was calculated using the New Injury Severity Score (NISS). Multivariable Cox proportional hazards regression modeling was used with results expressed as hazard ratios and 95 % confidence intervals.

**Results:**

Of the 914 children with at least one suspected NAT event, 39 % had at least one suspected recurrent NAT event; 12 % had 2 events and 5 % had ≥ 3 events during follow-up. Factors associated with an increased risk for a recurrent episode of suspected NAT with higher NISS were living in a rural area (1.69, 1.02–2.78, *p* = 0.04) and having an open wound (2.12, 1.24–3.62, *p* = 0.006), or superficial injury (2.28, 1.31–3.98, *p* = 0.004). In contrast, a greater number of injuries was associated with a decreased risk for a recurrent episode of suspected NAT with higher NISS (*p* < 0.0001).

**Conclusions:**

Though limited by a lack of follow-up of children placed in out of home care, our results suggest that children with “minor” or less numerous injuries are either not reported to child protective services or not removed from the unsafe environment with either situation leading to subsequent events. The medical and child welfare systems need to better identify these potential victims of recurrent events..

## Background

Non-accidental trauma (NAT) is a leading cause of injury and death throughout early childhood. In 2011, an estimated 686,000 (9.2 per 1000 children in the population) children across the United States were found to have substantiated or indicated cases of child maltreatment. An estimated 1,640 of these children died at a rate of 2.2 per 100,000 children in the population [1]. Many children who are victims of NAT may be repeatedly evaluated for injuries related to maltreatment. Past analysis of data from the National Child Abuse and Neglect Data System found that approximately one-third of children who are the subjects of first maltreatment reports are re-reported within 5 years. Of these children, nearly 17 % had one additional report and 11 % of children had multiple reports [2].

Children who are victims of recurrent NAT are at increased risk for mortality with each subsequent evaluation [3–5]. Several studies have demonstrated significantly higher mortality rates in abused children presenting with a recurrent episode of NAT compared to children presenting with an initial episode of NAT [6–8]. Previously reported risk factors associated with recurrent injury in victims of suspected NAT include younger age of the victim (<30 months) and initial presentation with “minor” injuries, such as dislocations, open wounds and superficial cutaneous injuries [9]. Despite these identified factors associated with recurrent episodes of NAT, little is known about how the severity of injury changes from event to event in children who survive recurrent events of suspected NAT and whether particular factors or injuries at one event are associated with a subsequent event with escalating severity of injury.

Identification of factors present at a suspected NAT event that are associated with subsequent NAT events of escalating severity of injury may help to identify children at the highest risk of subsequent serious injury. As resources to investigate cases of suspected NAT are limited, cases with a recurrent event of increasing severity should be the first patients to target for resources. The objective of this study is to identify risk factors for escalating severity of injury in children with multiple events of suspected NAT, using administrative claims data from a pediatric Medicaid accountable care organization.

## Methods

### Data source

Data was obtained from Nationwide Children’s Hospitals’ pediatric accountable care organization (ACO), Partners for Kids (PFK). PFK contracts with Medicaid managed care organizations to manage the care of almost 300,000 children in Central and Southeastern Ohio. Forty percent of the children in PFK live in Franklin County, the most urban county in the region, with the remainder spread throughout 33 other counties in Ohio, most of which are rural. During the study period, all children in PFK were enrolled through Ohio’s Covered Families and Children eligibility category for children from low-income families. The PFK claims database includes information on all billable medical care, procedures, and encounters for its enrollees, allowing for tracking of patients over time, across institutions, and across both inpatient and outpatient encounters. Data for this study was obtained by request from the PFK administration. The conduct of this study was approved by Nationwide Children’s Hospital Institutional Research Board with a waiver of informed consent.

### Study population

**Fig. 1:**
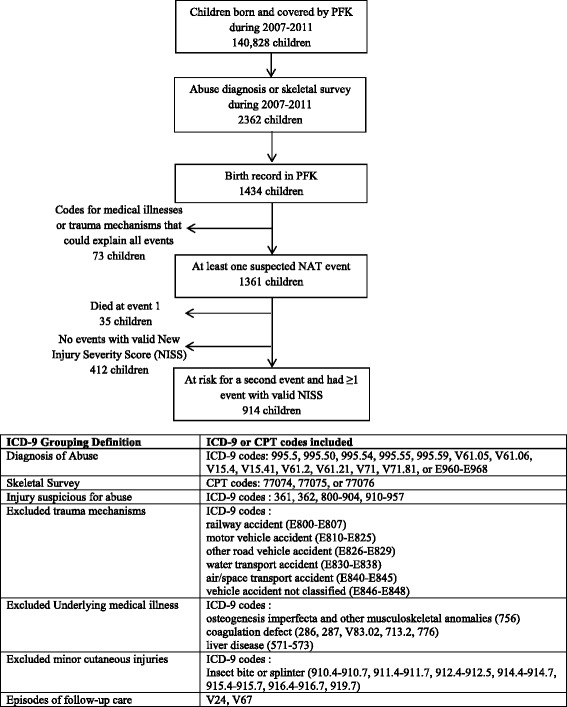
Determination of Study Population

Children with suspected NAT episodes were identified in the PFK database as previously described [9]. Briefly, children with birth claims and at least one non-birth related claim indicating a diagnosis of NAT or a skeletal survey from 2007 to 2011 were included (Fig. 1). Suspected NAT events were defined as episodes of care in which a claim contained either (a) an International Classification of Diseases, Ninth Revision, Clinical Modification (ICD-9-CM) discharge diagnosis code specific for child abuse, (b) a Current Procedural Terminology (CPT) coded skeletal survey, or (c) ICD-9 coded injuries suspicious for abuse; these events could be the event that brought the child into the study cohort, or they could occur either before or after that event. Events that had an ICD-9 E-code for a trauma mechanism that could explain the injury or an ICD-9 code for an underlying medical illness that could explain the injury or need for skeletal survey were excluded. We also excluded episodes of care with only a diagnosis of minor cutaneous injury from a specific mechanism, those coded as follow-up care, those with death at the first event and those without a valid new injury severity score. We defined an episode of care as encompassing all claims for service provided concurrently or within two days of the care documented in the claim in order to include all claims related to a single incident of suspected NAT. To minimize the risk of defining claims for follow-up care as new events, we only considered episodes of care for encounters in the emergency department, urgent care, or inpatient setting as recurrent events.

### Independent variables

Variables determined at the time of each event included age, sex, and days since the last suspected NAT event. The location, type, and mechanism of injuries, the number of injuries, and injury severity (evaluated as the new injury severity score (NISS)) [10] were also determined. The NISS was chosen because it allows for the consideration of multiple injuries in the same body region, unlike the injury severity score (ISS). In addition, in a recent systematic literature review, the NISS was found to be a better predictor of most clinical outcomes, including mortality and length of hospital stay, than the ISS [11]. Because family socioeconomic status (SES) indicators, such as parental education level and household income, were not available in the PFK database, zip code level SES variables such as percent of individuals living below poverty and urban vs. rural residence were determined from 2007 to 2011 American Community Survey 5-year estimates, based on each child’s zip code at his or her first event [12]. Age at each event and the zip code based SES variables were divided into a priori defined categories; age was divided into six month intervals and SES variables were divided at their medians.

### Statistical analysis

Characteristics at each suspected NAT event were summarized using descriptive statistics (medians and inter-quartile ranges (IQR) or frequencies and percentages). To define escalating and non-escalating injury severity from one event to the next event, the change in NISS between events was calculated. An escalating event was defined as a recurrent event with an increase in NISS of at least 1 category (i.e. NISS 1–3 (minor injury) to 4–75 (moderate, serious, severe, or critical); NISS 4–8 (moderate) to ≥9 (serious, severe, or critical), etc.), and a non-escalating event was defined as a recurrent event with a NISS of the same or lesser category as that of the previous event. Events that had either no valid nature of injury codes (N-codes) or no valid N-codes with both a known severity and known body region were classified as having unknown NISS and were not used in the determination of escalation in the severity of immediately following events.

To determine risk factors for recurrent events of escalating injury severity, we used an extension of the Cox proportional hazards model for recurrent event data, the Prentice, Williams and Peterson gap time (PWP-GT) model [13]. In these models, individuals were censored if they had no recurrent event or had a recurrent event with non-escalating or unknown change in injury severity. Due to low sample sizes for events beyond the first and second event, parameter estimates for all risk factors were assumed to be the same across all recurrences (first, second, third, or fourth recurrence), but the baseline hazard function was allowed to vary. Robust variance (sandwich) estimators were used to adjust for within-subject correlation in the time between events, which was the outcome in all models. Events beyond the fifth event were not considered in order to maintain a sufficient sample size for each event. Predictor variables in these models were the independent variables as measured at the previous event, with the exception of zip code based variables, which were determined based on the child’s address at the first event only.

Multivariable modeling was performed to determine the associations between individual variables and time to an escalating recurrent event. Risk factors present in < 5 children having a recurrent event were excluded. Individual factors associated at *p* < 0.20 with the time to either type of event in univariable models were included in a multivariable model. Variables were eliminated from this model in order of decreasing statistical significance until all variables in the model achieved a significance level of *p* < 0.05. In the final multivariable model, all predictors were checked for departure from the proportional hazards assumption and influential observations/outliers and goodness of fit were assessed [14, 15]. The final multivariable model revealed the overall associations of factors measured at any particular event with the risk of a subsequent event of escalating injury severity, after adjustment for other measured risk factors. In order to eliminate the possibility of missed events during enrollment breaks, a sensitivity analysis was performed in which all modeling was repeated including only those children who were continuously enrolled in PFK. Continuous enrollment was defined as no intermittent breaks in enrollment, but no limit was placed on the length of enrollment. Lastly, we examined the duration of enrollment of children in our cohort with events of severe or critical injury severity (NISS > 15) after such an event, in order to get a rough estimate of how many children might have been lost to follow-up when placed in out of home care. We further examined these patients in a sensitivity analysis by identifying risk factors for severe recurrent events (NISS > 15) regardless of the severity of the preceding event. All statistical analyses were performed using SAS (Statistical Analysis Software v9.3, SAS Institute, Cary, NC).

## Results

### Identification of cohort

During 2007–2011, PFK managed the health care of 140,828 children born during that time period. Of these children, 2362 had an abuse diagnosis code or skeletal survey code on a subsequent non-birth related claim. Sixty-one percent of these children had birth records in the PFK database (*N* = 1434; Fig. 1). After excluding events with diagnosis codes for a trauma mechanism or medical illness that could potentially explain the injury, 1,361 children had at least one incident of suspected NAT. Of these children, 35 died at their first event, and 412 had no events with a valid NISS, resulting in 914 patients in the study cohort. The 412 patients excluded for not having a NISS were not different from the included patients with regard to their demographic and socioeconomic characteristics (data not shown). Seventy-five percent (*N* = 687) of the final study cohort had an abuse diagnosis or skeletal survey at their first event.

### Population characteristics and injuries

Three hundred and sixty-one (39.5 %) of these children had more than one episode of care for suspected NAT during the study period (Table 1). The most common types of injuries across all events were contusions (28 % of events), fractures (27 % of events), open wounds (17 % of events), and superficial injuries (12 % of events) (Table 2). When all events were examined together, the most common type of contusion was a contusion of the head or neck, excluding the eye (*N* = 264, 50 % of contusions); the most common type of fracture was a skull fracture (*N* = 208, 30 % of fractures); the most common open wound was an open wound of the head, not including the ear or eye areas (*N* = 156, 55 % of open wounds); and the most common type of superficial injury was a superficial injury to the head or neck, excluding the eye (*N* = 97, 49 % of superficial injuries).

**Table 1:** Demographic characteristics by event

	Event 1	Event 2	Event 3	Event 4	Event 5
Number of children	914	361	111	37	15
Male, N (%)	496 (54.27)	203 (56.23)	59 (53.15)	24 (64.86)	10 (66.67)
Lives in urban area (at first NAT), N (%)^a^	670 (73.46)	256 (70.91)	75 (67.57)	23 (62.16)	7 (46.67)
Age, N (%)					
0–6 months	291 (31.84)	29 (8.03)	1 (0.9)	1 (2.7)	1 (6.67)
6–12 months	201 (21.99)	45 (12.47)	5 (4.5)	2 (5.41)	1 (6.67)
12–18 months	159 (17.4)	75 (20.78)	16 (14.41)	6 (16.22)	2 (13.33)
18–24 months	99 (10.83)	80 (22.16)	21 (18.92)	7 (18.92)	2 (13.33)
24–30 months	69 (7.55)	52 (14.4)	34 (30.63)	21 (56.76)	9 (60)
> 30 months	95 (10.39)	80 (22.16)	34 (30.63)	21 (56.76)	9 (60)
Percent of individuals living below poverty, median (IQR)^a^	19.75 (14.1, 26.6)	19.4 (14.1, 26.8)	19.7 (15.7, 26.8)	20.5 (18.2, 29.6)	19.2 (16.8, 22.5)
Dx type, N (%)					
Skeletal Survey	568 (62.14)	102 (28.25)	20 (18.02)	2 (5.41)	0 (0)
Abuse Code	332 (36.32)	87 (24.1)	21 (18.92)	10 (27.03)	2 (13.33)
Injury	730 (79.87)	308 (85.32)	97 (87.39)	33 (89.19)	15 (100)

^a^Based on the child’s zip code at their first suspected non-accidental trauma (NAT) event and based on 5-year averages from the 2007 to 2011 American Community Survey of the US Census

**Table 2:** Injury characteristics by event

	Event 1	Event 2	Event 3	Event 4	Event 5
Number of children	914	361	111	37	15
Injury type, N(%)					
Fracture	307 (33.59)	61 (16.9)	12 (10.81)	7 (18.92)	2 (13.33)
Dislocation	34 (3.72)	25 (6.93)	10 (9.01)	2 (5.41)	2 (13.33)
Burn	42 (4.6)	18 (4.99)	6 (5.41)	0 (0)	1 (6.67)
Retinal hemorrhage	29 (3.17)	10 (2.77)	0 (0)	0 (0)	0 (0)
Intracranial	81 (8.86)	18 (4.99)	3 (2.7)	3 (8.11)	2 (13.33)
Abdominal thoracic	19 (2.08)	4 (1.11)	1 (0.9)	0 (0)	0 (0)
Open wound	104 (11.38)	95 (26.32)	28 (25.23)	13 (35.14)	6 (40)
Superficial Injuries	114 (12.47)	44 (12.19)	13 (11.71)	6 (16.22)	2 (13.33)
Contusions	267 (29.21)	91 (25.21)	33 (29.73)	11 (29.73)	2 (13.33)
Other (Blood vessel, Crush, Spinal cord)	14 (0.02)	6 (0.02)	4 (0.04)	0 (0)	0 (0)
Location of injury, N (%)					
Head/neck	268 (29.32)	72 (19.94)	14 (12.61)	8 (21.62)	4 (26.67)
Face	64 (7)	25 (6.93)	10 (9.01)	1 (2.7)	3 (20)
Chest	59 (6.46)	13 (3.6)	3 (2.7)	1 (2.7)	1 (6.67)
Abdomen and pelvic contents	47 (5.14)	14 (3.88)	2 (1.8)	3 (8.11)	0 (0)
Extremities or pelvic girdle	337 (36.87)	104 (28.81)	35 (31.53)	10 (27.03)	3 (20)
External	509 (55.69)	218 (60.39)	78 (70.27)	21 (56.76)	8 (53.33)
Number of injuries, N (%)					
0	76 (8.32)	37 (10.25)	8 (7.21)	3 (8.11)	0 (0)
1	279 (30.53)	138 (38.23)	41 (36.94)	17 (45.95)	7 (46.67)
2	218 (23.85)	103 (28.53)	38 (34.23)	10 (27.03)	5 (33.33)
3	90 (9.85)	31 (8.59)	16 (14.41)	2 (5.41)	2 (13.33)
4	67 (7.33)	19 (5.26)	2 (1.8)	3 (8.11)	0 (0)
5+	184 (20.13)	33 (9.14)	6 (5.41)	2 (5.41)	1 (6.67)
Died during episode, N (%)	0 (0)	2 (0.55)	0 (0)	0 (0)	0 (0)
New Injury Severity Score, median (IQR)	4 (1, 12)	2 (1, 5)	2 (1, 5)	2 (1, 5)	2 (1, 5)

### Characterization of recidivism

**Fig. 2:**
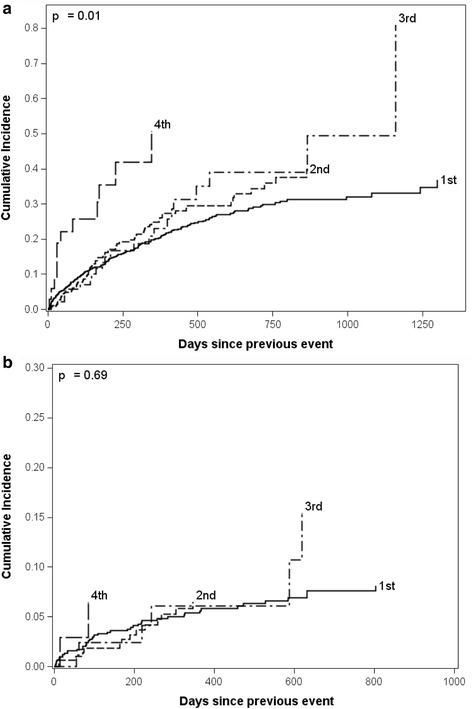
Kaplan-Meier curves for the cumulative incidence of recurrent events of (**a**) non-escalating injury severity and (**b**) escalating injury severity. The proportion of at risk patients that have a recurrent event (y-axis) over time since their previous event (x-axis) is displayed. Recurrent events occurred at a significantly greater rate with increasing event number in episodes with non-escalating injury severity (Panel **a**; *p* = 0.01), but not in episodes with escalating injury severity (Panel **b**; *p* = 0.69)

Among those children with multiple events during the study period, the median time between the first and second events was 191 days (IQR 71, 393). The median NISS was on average 1.6 times higher at the first event than at recurrent events (*p* < .0001). Kaplan-Meier survival analysis estimated that 34.8 % of the children had ≥1 recurrent event within 1 year of their initial event and 52.8 % had ≥1 recurrence within 2 years of their initial event. Of the children who had ≥ 1 recurrent event, 33.9 % had subsequent recurrence within 1 year of their first recurrence. When recurrent episodes of suspected NAT were examined based on changes in severity between events (escalating NISS or non-escalating NISS), recurrent events of non-escalating injury severity were found to occur at much higher rates than recurrent events of escalating injury severity (*p* < 0.05) In addition, recurrent events of escalating injury severity occurred at similar rates across all recurrent events (*p* = 0.69), but recurrent events of non-escalating injury severity occurred at a significantly greater rate with increasing event number (*p* = 0.01) (Fig. 2).

### Risk of recidivism

In bivariate analyses, factors associated with having a lower risk for a subsequent event of escalating injury were: having a fracture, a head or neck injury, an injury to the extremities or pelvic girdle, having two or more injuries, and living in a zip code with a higher poverty rate (Table 3). Children with open wounds or superficial injuries had a greater risk of having a subsequent event of escalating severity (*p* < 0.05 for all) (Table 3). In multivariable models, factors independently associated with an increased risk for a recurrent episode of suspected NAT with higher NISS were living in a rural area (HR 1.69, 95 % CI 1.02–2.78, *p* = 0.04) and having an open wound (2.12, 1.24–3.62, *p* = 0.006), or superficial injury (2.28, 1.31–3.98, *p* = 0.004) (Table 4). In contrast, having a greater number of injuries was associated with a decreased risk for a recurrent episode of suspected NAT with higher NISS (*p* < 0.0001). Compared to patients with ≤ 1 injury, patients with 2–3 injuries had a hazard ratio for a more severe recurrent episode of NAT of 0.40 (0.24–0.67) and patients with ≥4 injuries had a hazard ratio of 0.11 (0.04–0.31) (Table 4).

**Table 3:** Univariable Cox model for recurrent suspected NAT with escalating severity of injury

Variable	HR	(95 % CI)		*P*
Male	1.119	0.702	1.784	0.637
Lives in rural area^a^	1.513	0.933	2.453	0.093
Age				
0–6 months	ref			0.107
6–12 months	1.707	0.789	3.693	
12–18 months	2.346	1.098	5.010	
18–24 months	1.877	0.803	4.386	
24–30 months	2.277	0.918	5.649	
> 30 months	0.764	0.254	2.303	
Injury type, N(%)				
Fracture	0.201	0.087	0.466	0.0002
Dislocation	0.885	0.286	2.743	0.833
Burn	0.667	0.094	4.739	0.686
Intracranial	0.169	0.023	1.227	0.079
Open wound	2.041	1.206	3.452	0.008
Superficial Injuries	1.945	1.124	3.367	0.017
Contusions	1.123	0.681	1.850	0.650
Location of injury				
Head/neck	0.347	0.174	0.692	0.003
Face	1.206	0.525	2.772	0.659
Chest	0.724	0.423	1.239	0.239
Abdomen and pelvic contents	0.506	0.120	2.140	0.355
Extremities or pelvic girdle	0.500	0.287	0.872	0.015
External	1.336	0.786	2.270	0.284
Number of injuries				
0–1	ref			<.0001
2–3	0.408	0.242	0.687	
4+	0.127	0.045	0.352	
Percent of individuals living below poverty in patient’s zip code > cohort median of 19.75%^a^	0.612	0.380	0.987	0.044

^a^Based on the child’s zip code at their first suspected non-accidental trauma (NAT) event and based on 5-year averages from the 2007 to 2011 American Community Survey of the US Census

**Table 4:** Multivariable Cox model for recurrent suspected nat with escalating severity of injury

Entire Cohort				
Variable	HR	(95 % CI)		*P*
Lives in rural area^a^	1.69	1.02	2.78	0.04
Open wound injury	2.12	1.24	3.62	0.01
Superficial injury	2.28	1.31	3.98	0.004
Number of injuries				
0–1	ref			<.0001
2–3	0.40	0.24	0.67	
4+	0.11	0.04	0.31	
Continuously enrolled children only				
Lives in rural area^a^	2.01	1.03	3.91	0.04
Fracture	0.23	0.07	0.76	0.02
Open wound injury	2.30	1.14	4.66	0.02

^a^Living in a rural area was based on the child’s zip code at their first event. All other predictor variables in these models were the values of the independent variables at the previous event

### Sensitivity analysis in patients with continuous enrollment

When analyses were repeated including only those children who maintained continuous enrollment in the PFK for at least 2 years (*N* = 576, 63.0 %), the 1-year and 2-year recidivism rates were 30.6 and 42.4 % respectively for the first recurrence, and the recidivism rate for a second recurrence within 1 year of the first was 28.5 %. In multivariable modeling, having a fracture was predictive of a lower risk for a subsequent event of increased NISS (Table 4), whereas living in a rural area and having an open wound continued to be predictive of an increased risk for subsequent suspected NAT of increased injury severity. Having a superficial injury and having fewer injuries were no longer significant predictors of the risk for subsequent suspected NAT of increased injury severity in this subgroup.

### Sensitivity analysis examining risk factors for any recurrent severe event

Because any recurrent severe NAT event, defined in this study as NISS > 15, would be of great concern, regardless of the severity of preceding events, we also examined risk factors for this type of occurrence. There were only 34 severe events in the study cohort. In multivariable models, factors independently associated with an increased risk for a recurrent severe NAT event were living in a rural area (HR 2.59, 95 % CI 1.26–5.31, *p* = 0.01) and having an intracranial injury (HR 3.04, 95 % CI 1.29–7.16, *p* = 0.01). In children with severe events, the duration of enrollment after such an event was actually quite long, with the median follow-up after such events being 460 days (IQR 188–807).

## Discussion

Many children who are victims of NAT may not experience abuse as a one-time event, but rather as a recurrence that is part of the high-risk environment in which they live. This is the first study, to our knowledge, to use administrative claims data from a pediatric Medicaid accountable care organization to identify risk factors for escalating severity of injury in children with multiple events of suspected NAT. In this study, factors predictive of an increased risk for more severe subsequent episodes of suspected NAT include living in a rural area, having an open wound and having a superficial injury. Conversely, having more injuries is predictive of a decreased risk for a subsequent episode of suspected NAT of increasing severity.

Population-based studies and analyses of large datasets are becoming increasingly important in studying recurrent NAT. Friedlaender et al. analyzed system-level Medicaid claims data to characterize the health service use patterns of maltreated children in the year before their first reported episode of maltreatment [16]. The authors demonstrated that victims of maltreatment changed ambulatory care providers with greater frequency than those children who were not abused. The study design, however, did not allow for the study of recurrence of abuse nor identification of specific patterns or types of injuries that place a child at increased risk for recurrent maltreatment. Schmitt et al. studied a population of abused children who were returned to the home in which the abuse occurred and found that these children had a higher risk of a fatal recurrent episode of 5–10 % [6]. Similarly, Putnam-Hornstein et al. prospectively studied a population of over four million children following a nonfatal allegation of maltreatment [7]. Findings from this study indicate that after adjusting for risk factors at birth, children with a prior allegation of maltreatment died from intentional injuries at a rate that was 5.9 times greater than unreported children (95 % CI [4.39, 7.81]). In a previous analysis, we demonstrated that child victims of recurrent abuse had significantly higher mortality rates compared to victims of a single episode of abuse (24.5 % vs. 9.9 %; *p* = .002) [8]. We also have previously reported on risk factors associated for recurrent injury in victims of suspected NAT, including young age of the victim (<30 months) and initial presentation with “minor” injuries, such as dislocations, open wounds and superficial cutaneous injuries [9]. Our present study adds to the existing literature by identifying factors associated with children experiencing recurrent episodes of NAT of increasing severity. This type of data may help health care providers to develop screening protocols to identify children at the highest risk for subsequent serious injury at their initial episode.

Our findings highlight a potential bias in the identification and diagnosis of injury by the healthcare provider, or in the subsequent child protective services response to children who present with “minor”, or less numerous injuries. It seems likely that children with “minor” or less numerous injuries are either not reported to child protective services by the healthcare provider or not removed from the unsafe environment despite a report being made, with either situation leading to subsequent events. Sheets et al. report that nearly one in three children evaluated for abuse were seen previously with a sentinel injury, the vast majority of which were simply bruises [17]. Our data demonstrate that not only are these children more likely to experience subsequent events, but identifies specific risk factors that are predictive of increasing severity across recurrent events. Likewise, our finding that children living in rural areas are at increased risk for escalating severity raises the idea that resource scarcity may be a concern, either with access to medical expertise to identify injuries or with the ability of the child welfare system to respond appropriately to concerns.

There are several limitations related to using system-level administrative claims data in this study. First, approximately 35 % of patients had at least one break in Medicaid enrollment during the study period with the potential that recurrent events could have occurred during the time of non-enrollment; therefore our data may be an underestimate of recurrent events. However, in a sensitivity analysis excluding children with discontinuous enrollment during the study period, the findings were similar, though the number of injuries and having a superficial injury no longer reached statistical significance in this subgroup. Second, as with any study utilizing claims data, we are limited in the sensitivity and specificity of the ICD-9 coding practices used to identify key variables. Specifically, ICD-9 coding from an admission may underestimate the prevalence of abusive injuries as physicians may be reluctant to assign a diagnosis of NAT without confirmation of the mechanism as child abuse from a multi-disciplinary investigation that is usually not complete until after discharge. Third, administrative datasets provide limited data on covariates of interest including race, parental characteristics, and SES characteristics. Lastly, this study lacked information on children removed from the home after suspected NAT events. Most children covered by PFK who go into child protective services custody are moved to fee-for-service Medicaid and thus no longer covered by PFK, though this varies by county in Ohio. Therefore, we were unable to follow the majority of patients who might have been placed in out of home care. Medicaid Analytic Extract (MAX) files might be useful to track these patients, in that Medicaid IDs could be used to follow children from PFK into fee-for-service Medicaid. However, we did not have access to this data for this study. The above limitations were unavoidable; however they likely resulted in under-identification of suspected NAT events with minimization rather than exaggeration of our findings. Although the last noted limitation could have biased our findings towards the identification of characteristics prevalent in children not removed from the home after suspected NAT, we were able to follow the majority of children who experienced severe events for more than 1 year after such an event

## Conclusion

This study used administrative claims data from a pediatric Medicaid accountable care organization to identify factors associated with escalating severity of injury in children with multiple events of suspected NAT. Factors associated with escalating severity in this study include living in a rural area and having “minor” injuries such as an open wound or superficial injury. Conversely, having more injuries is associated with a decreased risk for more severe subsequent events. These findings identify potential biases and/or resource issues in the response of the medical and child welfare systems to children who present with less severe injuries and suggest that these systems need to better identify potential victims of abuse presenting with minor injuries to prevent subsequent, more severe episodes of potential NAT. Future steps may require increased resources but could include raising awareness through education that when treating minor injuries in young children, abuse should always be considered because of the risk for recurrent events with worse injuries. In addition, a systematized process to screen for abuse in injured young children (e.g. a screening tool) should be considered by all providers treating children.

## Abbreviations

CPT: Current procedural terminology
HR: Hazard ratio
ICD-9-CM: International Classification of Diseases, Ninth Revision, Clinical Modification
NAT: Non-accidental trauma
PFK: Partners for kids
PWP-GT: Prentice, Williams and Peterson gap time model
SES: Socioeconomic status
TMPM-ICD9: Trauma mortality prediction model
